# Supplementary material for: Bacterial and Metabolic Factors of Staphylococcal Planktonic and Biofilm Environments Differentially Regulate Macrophage Immune Activation
Source: Inflammation. 2023 May 22;46(4):1512–30. doi: 10.1007/s10753-023-01824-3 (PMC10359233; doi:10.1007/s10753-023-01824-3)
Supplement: Supplementary file 1 — Supplementary material accompanies the manuscript, which is available in a separate file. The non-revised manuscript is available online in the pre-print version https://doi.org/10.1101/2021.07.26.453923 (DOCX 1578 KB) [file 10753_2023_1824_MOESM1_ESM.docx]

**Supplementary material**

**Paper: Bacterial and metabolic factors of staphylococcal planktonic and biofilm environments differentially regulate macrophage immune activation**

**by Elisabeth Seebach, Tabea Elschner, Franziska V. Kraus, Margarida Souto-Carneiro and Katharina F. Kubatzky**

**
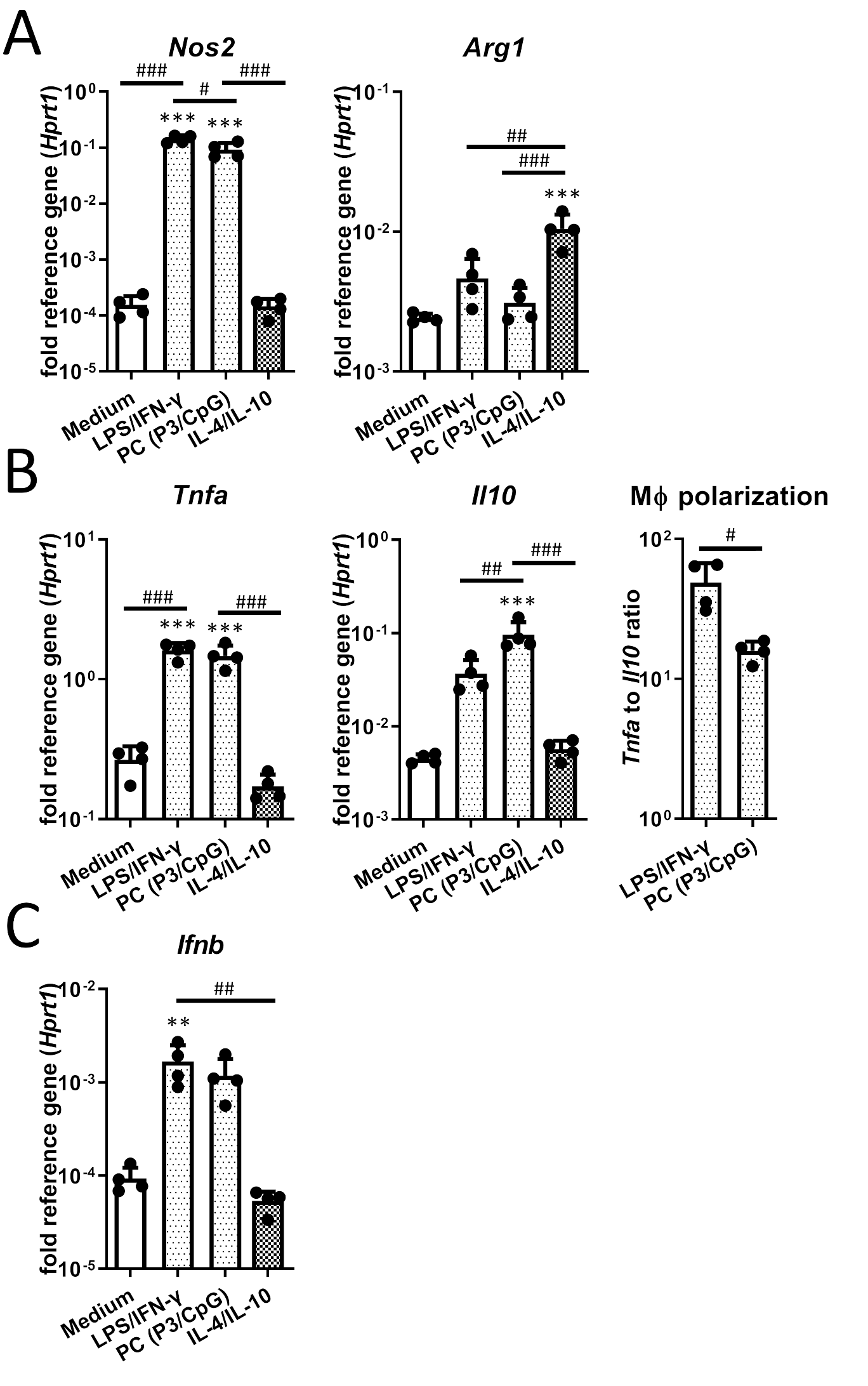
**

**Suppl. Fig. 1** Macrophage M1/M2 polarization and validation of related gene expression. RAW 264.7 cells were stimulated with 50 ng/ml LPS and 20 ng/ml IFN-γ (M1), PC (positive control: 1 µg/ml Pam3CSK4 and 100 nM CpG ODN) or IL-4 and IL-10, both 20 ng/ml (M2). After 20 hours of stimulation, gene expression levels were analyzed. Gene expression analysis of marker genes *Nos2* (M1) and *Arg1* (M2) (A), pro-inflammatory *Tnfa* and anti-inflammatory *Il10* (B) and *Ifnb* (C). Ratio of *Tnfa* to *Il10* expression levels was used as indicator for macrophage polarization. Data are presented as relative gene expression of gene of interest related to the reference gene *Hprt1*. n=4 experiments. Mean + SD are shown with single values as dots. p-values are calculated by Ordinary one-way ANOVA with post-hoc Bonferroni corrected multiple comparison. Asterisk is indicating significance against Medium, number sign is showing significance between respective treatment groups. * p<0.05, ** p<0.01, *** p<0.001; # p<0.05, ## p<0.01, ### p<0.001.


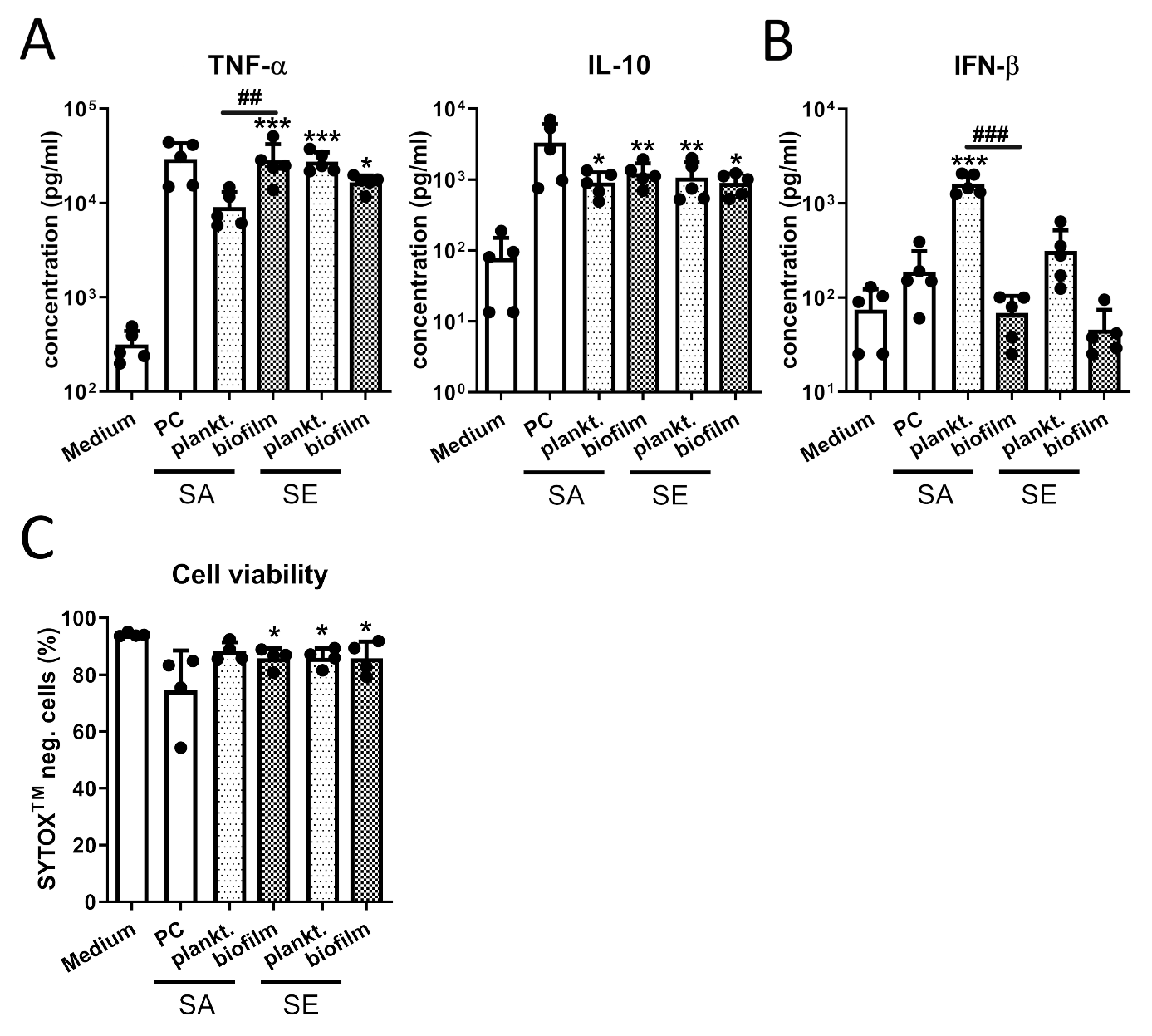


**Suppl. Fig. 2** Protein concentrations of cytokines released by macrophages and cell viability upon stimulation with CM. RAW 264.7 cells were cultivated in CM 1:1 diluted in fresh growth media (DMEM high glucose + 10% FCS + 1% Pen/Strep) and cytokine release was analyzed. Cells were stimulated with CM for 20 hours and concentrations of pro-inflammatory TNF-α and anti-inflammatory IL-10 (A) as well as IFN-β (B) were quantified in the supernatant by cytometric bead array (CBA; LEGENDplex^TM^). Data are presented as absolute concentration (pg/ml). n=5 experiments. C) Viability of macrophages after CM stimulation. Cells were stimulated with CM for 20 hours and cell viability was measured by SYTOX^TM^-staining and FACS analysis. Percentage of SYTOX^TM^-negative (living) cells are shown. n=4 experiments. For all: Mean + SD are shown with single values as dots. p-values are calculated by Ordinary one-way ANOVA with post-hoc Bonferroni corrected multiple comparison. Asterisk is indicating significance against Medium, number sign is showing significance between respective planktonic and biofilm CM. * p<0.05, ** p<0.01, *** p<0.001; # p<0.05, ## p<0.01, ### p<0.001. PC: positive control (1 µg/ml Pam3CSK4 + 100 nM CpG ODN).


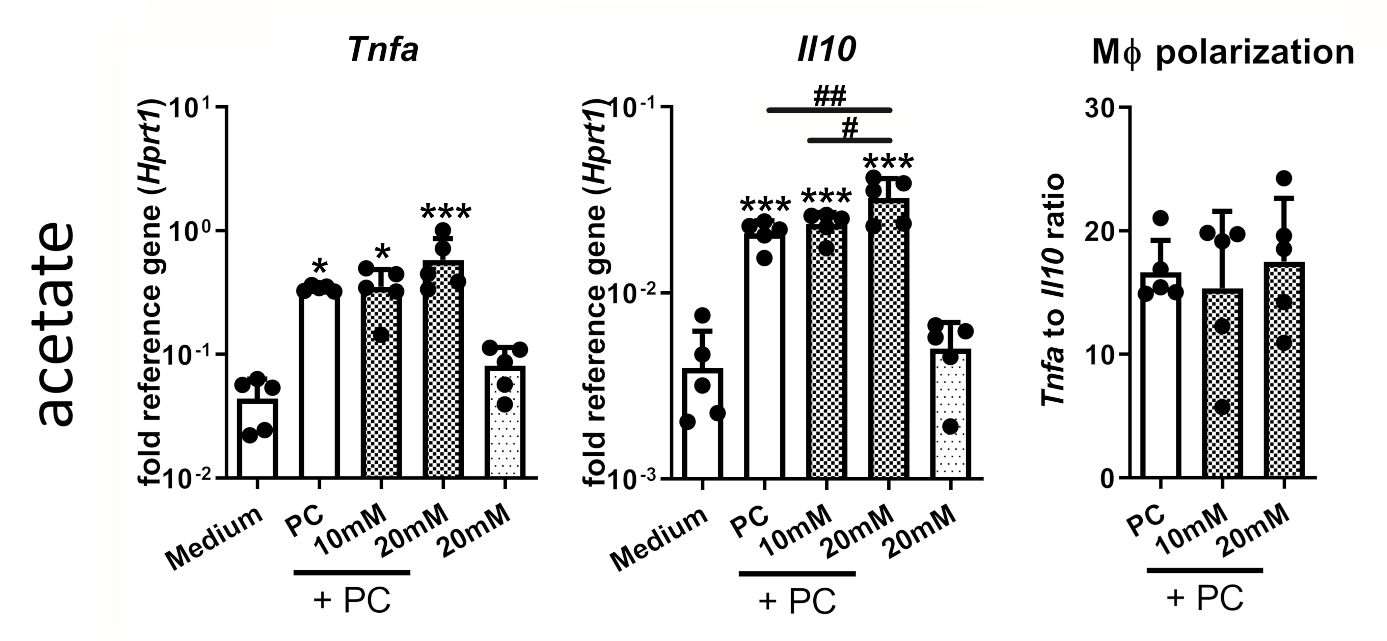


**Suppl. Fig. 3** Effect of bacteria-derived acetate on macrophage TLR-2/-9 response. RAW 264.7 cells were stimulated with PC (positive control: 1 µg/ml Pam3CSK4 + 100 nM CpG ODN) for 20 hours with different extracellular acetate concentrations (10 and 20 mM) added to the medium and immune response was evaluated. Gene expression analysis of pro-inflammatory *Tnfa* and anti-inflammatory *Il10*. Ratio of *Tnfa* to *Il10* expression levels was used as indicator for macrophage polarization. Data are presented as relative gene expression of gene of interest related to the reference gene *Hprt1*. n=5 experiments. Mean + SD are shown with single values as dots. p-values are calculated by Ordinary one-way ANOVA with post-hoc Bonferroni corrected multiple comparison. Asterisk is indicating significance against Medium, number sign is showing significance between acetate concentrations. * p<0.05, ** p<0.01, *** p<0.001; # p<0.05, ## p<0.01, ### p<0.001.
